# Supplementary material for: Used economy market insight: Sailboat industry pricing mechanism and regional effects
Source: PLoS One. 2025 Jan 17;20(1):e0315101. doi: 10.1371/journal.pone.0315101 (PMC11741610; doi:10.1371/journal.pone.0315101)
Supplement: Attachment — Submitted filename: Response to Reviewer 2 Comments.docx [file pone.0315101.s001.docx]

**Response to Reviewer 2 Comments**

Dear reviewer,

We sincerely thank you for examining our manuscript and providing helpful comments to guide our revision. We have tried to revise the manuscript according to your kind comments and suggestions. Please find the following detailed responses to your comments and suggestions. Finally, we would like to thank the referee again for taking the time to review our manuscript.

1. There is little introduction to the dataset, and it is recommended to strengthen the introduction to the amount and structure of the data set. At the same time, data preprocessing is also a key part of the narrative, and more space should be devoted to issues such as data cleaning.

**Response 1:** Thank you for your suggestions.In response to your suggestion, we have significantly strengthened the introduction to our dataset. The dataset now covers two types of sailboats: Monohulled Sailboats and Catamarans. The key variables include listing price, brand, model, length (in feet), geographic region, country/region/state, production year, economic level, sail area, displacement, stability, comfort, headroom, and draft. The dataset spans production years from 2005 to 2019, with a total of 2,346 Monohulled Sailboats and 1,145 Catamarans samples. Moreover, we have restructured the dataset into subsets categorized by sailboat type and geographical region. This reorganization enables us to conduct a more granular analysis, focusing on the dimensions of sailboat type and regional effects.

2. The article proposes using a hybrid model based on RF-XGB for selecting important indicators, and provides specific calculation indicators to illustrate the advantages of this method. However, it does not analyze the advantages of the model for this problem from the perspective of the model's own principles, and lacks theoretical support. It is recommended that this section be supplemented.

**Response 2:** Thank you for your suggestions.

Theoretical Support for the Model

Ensemble learning theory posits that combining multiple learners can significantly enhance a model's generalization ability. The hybrid use of Random Forest (RF) and XGBoost, two well-regarded ensemble learning algorithms, is consistent with the core principles of ensemble learning and offers a powerful approach to predictive modeling.

Model fusion techniques, such as Stacking, Bagging, and Boosting, are widely recognized for their effectiveness in improving model performance. The combination of Random Forest and XGBoost can be viewed as a specialized form of model fusion, designed to capitalize on the strengths of both algorithms to achieve superior predictive results.

The integration of Random Forest and XGBoost models provides unique advantages, particularly in handling complex datasets and tasks that require a more nuanced approach.

Firstly, the hybrid model enhances generalization capabilities. Random Forest constructs multiple decision trees using bootstrap sampling and random feature selection, introducing randomness that helps mitigate overfitting and increases the model's robustness. XGBoost, employing gradient boosting, incrementally refines the model to capture complex nonlinear relationships, often resulting in higher predictive accuracy. By combining these two approaches, the model leverages Random Forest's robustness to counterbalance any potential overfitting that might occur with XGBoost, while simultaneously utilizing XGBoost's precision to improve overall predictive performance.

Secondly, the hybrid model allows for a more comprehensive evaluation of feature importance. Random Forest provides a straightforward mechanism for assessing the contribution of each feature to the model’s predictions, which is invaluable for feature selection and model interpretation. XGBoost also offers feature importance metrics, though its evaluation approach differs slightly due to its underlying mechanisms. The combination of both methods offers a more complete perspective on data analysis and feature engineering, enabling a deeper understanding of the factors driving model performance. Additionally, the hybrid model is well-suited to managing diverse data distributions and noise levels. Random Forest's inherent randomness gives it a natural resilience to noise and outliers, while XGBoost’s use of regularization and adjustable learning rates allows for fine-tuning model complexity to better accommodate different data distributions and noise conditions. This flexibility enables the hybrid model to dynamically adjust the contributions of Random Forest and XGBoost based on the specific characteristics of the dataset, achieving an optimal balance between robustness and accuracy.

Moreover, the hybrid model benefits from the computational efficiencies of parallel processing and optimized training. XGBoost’s support for parallel computation significantly accelerates the training process, while the inclusion of Random Forest ensures that the model maintains strong predictive performance across a range of scenarios.

In conclusion, the combined advantages of the RF-XGB hybrid model, which are challenging to replicate with a single algorithm, make it a particularly promising approach for practical applications. This hybrid approach is especially well-suited to the research requirements of this study, which focuses on the analysis of second-hand sailboat market data.

3. The article proposes using NAR dynamic neural networks for price prediction, and the training of this model is also an important process that requires enhanced narration, such as strengthening the narration of the selection of model training parameters and providing analysis processes such as the trend of changes in the loss function.

**Response 3:** Thank you for your suggestions.

This study use of the Nonlinear Autoregressive (NAR) dynamic neural network for price prediction is indeed a critical component of this study.

Firstly, the selection of training parameters for the NAR model is crucial to ensuring its effectiveness and accuracy. In this study, we have carefully chosen key parameters based on both theoretical considerations and empirical testing. Specifically, we selected 10 neurons for the hidden layer and set the autoregressive order to 2. These choices were made to balance the model's complexity with its ability to capture the underlying patterns in the data, thereby avoiding overfitting while ensuring robust predictions. However, I recognize the importance of further elaborating on the rationale behind these selections, which I will include in the revised manuscript.

Additionally, the analysis of the loss function's behavior during training is an essential aspect that reflects the model's learning process. To address this, I will enhance the discussion by including a more detailed narration of the loss function's trend throughout the training epochs. This analysis, represented in Figure 7, provides insights into how the model's predictions improve as the loss function decreases, highlighting the convergence behavior and the model's optimization trajectory. By incorporating this explanation, readers will gain a clearer understanding of how the model's performance evolves during training and how the chosen parameters contribute to its stability and accuracy.

In summary, we have strengthen the manuscript by elaborating on the selection of training parameters for the NAR dynamic neural network and providing a more detailed analysis of the loss function's trend during the training process.

4. In the rolling price forecast section, the image interpretation is not clear, and the meaning of the Y axis is not clear, making the image fitting effect less accurate and less convincing.

**Response 4:** Thank you for your suggestions. We have clarified the meaning of the y-axis in the revised version to enhance the clarity and persuasiveness of the figure.

Considering that prices in the secondary market are prone to fluctuations due to market supply and demand, we have innovatively improved the delay order by adopting a rolling dynamic forecasting model that predicts future prices by rolling the price levels of previous periods.

In the double-hull sailboat NAR dynamic forecasting model, rolling forecasts begin from the 101st period, using data from the 1st to the 100th period to predict the pricing level for the 101st period, and then using data from the 2nd to the 101st period to predict the pricing level for the 102nd period, and so on. For the single-hull sailboat NAR dynamic forecasting model, due to the larger sample size, we set the forecasts to start rolling every 200 periods, with each forecast's results impacting subsequent results in a rolling manner—this is an innovative aspect of the model.

In Figure 8, the x-axis represents the number of predictions made by the neural network model, while the y-axis represents the market prices of used sailboats, including both actual market prices and predicted prices, with units in ×10^5 USD. From Figure 8, we observe that the actual prices of used sailboats exhibit strong fluctuations, while the forecasted values better reflect the overall price trend, with actual prices generally fluctuating around the forecast value. This provides a good reference for predicting the average level of future prices.

5. The article format needs to be changed, such as the incorrect arrangement of formulas

**Response 5:** We appreciate your feedback regarding the article format. We have made the necessary modifications to ensure that the arrangement of formulas is correct and adheres to the required formatting standards. Thank you for bringing this to our attention.

6. the following studies were recommended to be properly cited:

[1] Autonomous port management based AGV path planning and optimization via an ensemble reinforcement learning framework," Ocean & Coastal Management, vol. 251, p. 107087, 2024/05/01/ 2024, doi: https://doi.org/10.1016/j.ocecoaman.2024.107087.
[2] Ship energy consumption analysis and carbon emission exploitation via spatial-temporal maritime data," Applied Energy, vol. 360, p. 122886, 2024/04/15/ 2024, doi: https://doi.org/10.1016/j.apenergy.2024.122886.

**Response 6:** Thank you for your valuable feedback. We have added citations for the following studies as recommended.
