# Supplementary material for: Used economy market insight: Sailboat industry pricing mechanism and regional effects
Source: PLoS One. 2025 Jan 17;20(1):e0315101. doi: 10.1371/journal.pone.0315101 (PMC11741610; doi:10.1371/journal.pone.0315101)
Supplement: Attachment — Submitted filename: Response to Reviewer 3 Comments.docx [file pone.0315101.s002.docx]

**Response to Reviewer 3 Comments**

Dear reviewer,

We sincerely thank you for examining our manuscript and providing helpful comments to guide our revision. We have tried to revise the manuscript according to your kind comments and suggestions. Please find the following detailed responses to your comments and suggestions. Finally, we would like to thank the referee again for taking the time to review our manuscript.

1. This paper focuses on investigating the key influencing factors for price fluctuation in the second-hand sailboat. Sounds like an interesting topic.
**Response 1:** Thank you for your positive feedback on our research topic. We appreciate your recognition of the importance of investigating the key influencing factors for price fluctuations in the second-hand sailboat market. This area holds significant relevance, especially in the context of the growing circular economy. We aim to provide valuable insights that can contribute to both academic discourse and practical applications in the industry.

2. This study employs statistical analysis to investigate the key price influencing factors, and builds a multiple linear regression model for future price forecast via AI regression.
**Response 2:** Thank you for your comment. In our study, we employed statistical analysis to investigate the key factors influencing price fluctuations and established a multiple linear regression model for future price forecasting through AI regression. We utilized advanced techniques such as the random forest model and XGBoost (RF-XGB) to effectively pinpoint the core price indicators.

Additionally, we recognized the limitations of traditional linear regression models in capturing the complexities of price dynamics. Therefore, we used an innovative rolling Nonlinear Auto-Regressive (NAR) dynamic neural network model for more accurate price forecasting. This approach allows us to account for non-linear relationships and temporal dependencies in the data, which are critical in understanding the price movements of second-hand sailboats.

Furthermore, we constructed a regional effect multi-level model (RE-MLM) to examine how geographical, economic, and cultural factors contribute to pricing variations across different regions. This comprehensive methodology not only enhances the robustness of our findings but also provides a more nuanced understanding of the second-hand sailboat market. We believe that our approach can serve as a valuable framework for future research and practical applications in pricing strategies within this industry. Thank you for your insightful feedback, which encourages us to further elaborate on our methodologies and findings.

3. The paper is well written but difficult to follow. A sample price over time is good here.

**Response 3:** Thank you for your feedback regarding the clarity of our paper. We appreciate your acknowledgment of the quality of writing, and we understand that the complexity of the subject matter can make it challenging to follow.

To address this concern, we have incorporated a sample price trend over time in the revised manuscript (See Table 9). This addition will help illustrate the fluctuations and patterns in second-hand sailboat prices more clearly, providing readers with a visual representation that complements our statistical analysis.

4. A logical reason and trend is much needed in determining a predicting future price. An AI model on the past data does not give significant insight on how the price move in the near future.

Response 4: Thank you for your insightful comment regarding the need for a logical rationale and trend analysis in predicting future prices. We recognize that relying solely on an AI model based on historical data may not provide sufficient insight into future price movements.

In response to this, we have included a macro-level analysis of the development trends in the second-hand market following our price predictions. This analysis is grounded in the broader global economic context and relevant policies related to the second-hand economy, providing a logical framework that supports and validates our forecasting results.

We approached this macro analysis from four key perspectives:

(1) Market Supply and Demand: The increasing popularity of recreational sailing activities has driven demand for second-hand sailboats. Given the long production cycles and high costs associated with new boats, supply remains relatively stable. Consequently, as market demand rises, we anticipate an increase in second-hand sailboat prices.

(2) Global Economic Environment: Currently, the global economy is experiencing a downturn, leading to decreased consumer confidence and weakened demand, which may result in lower prices in the short term.

(3) National Policies: Many countries are placing greater emphasis on environmental sustainability. In the U.S., some states have implemented tax incentives for the purchase of second-hand boats to stimulate consumption. In Europe, initiatives like the UK's Green Boating Plan (launched in 2018) and France's Marine and Coastal Law (introduced in 2020) promote second-hand boat transactions to encourage sustainable practices.

(4) Technological Advancements: The emergence of new materials and designs for sailboats may lead to depreciation of older models. However, this also expands the options available in the second-hand market, potentially attracting more buyers.

In summary, our comprehensive analysis indicates that factors such as market dynamics, economic conditions, policy initiatives, and technological innovations will influence second-hand sailboat prices, which are expected to remain relatively stable or experience short-term fluctuations. Overall, as global interest in sustainable consumption and recreational activities continues to grow, we remain optimistic about the future of the second-hand sailboat market.

5. A geographical map will be good here. All the variable given in the model are not directed to the ships themselves and its condition.
**Response 5:** Thank you for your valuable feedback regarding the inclusion of a geographical map and the need for a clearer focus on the variables related to the ships themselves.

In our revised manuscript, particularly in section 3.2.2, we have added a comprehensive explanation of the variables used in our model to enhance clarity and presentation. This includes a more detailed discussion of the specific ship-related variables, ensuring that our analysis is more targeted and relevant to the subject matter.

In constructing our model, we included not only macroeconomic and geographical indicators but also specific metrics related to the sailboats themselves. These include the type, brand, model, length, sail area, displacement, stability, comfort, freeboard, and draft of the vessels. Please refer to Table 4 for a detailed breakdown of these indicators. Additionally, our performance metrics encompass evaluations of the sailboat's equipment and materials, including the mainsail, mast, port and starboard sides, rudder, and engine.

6. A final result on the accuracy of this model and its performance for the whole year 2023 is refreshing here.

**Response 6:** Thank you for your valuable suggestion regarding the inclusion of a final result on the accuracy and performance of our model for the entire year of 2023. In response to the request for a final result on the accuracy of our model and its performance for the year 2023, we have conducted a comprehensive evaluation of the predictive capability of our multilevel model, specifically focusing on the pricing of used sailboats across different regions.

Throughout 2023, the model demonstrated a robust predictive accuracy, maintaining a high degree of consistency with actual market prices. The predicted average prices for each geographic region—Caribbean, Europe, and the USA—were closely aligned with the observed values, thereby reinforcing the model’s effectiveness in capturing regional pricing dynamics.

For instance, the predicted prices in the Caribbean for 2023 were $504,484.99, while the actual observed prices showed only a marginal deviation from this estimate. Similarly, the model accurately predicted prices in Europe at $742,444.93 and in the USA at $939,290.63, both of which reflect minor variances from the actual market figures.

7. Authors have used many generic term without referring to the object which is the ship element for instance the intercept.

**Response 7:** Thank you for your careful review and valuable feedback. In response to your comment regarding the use of generic terms without specifically referencing ship elements, we have made detailed revisions to the manuscript.

We have incorporated specific descriptions of the ship-related variables to ensure that the terminology used is more precise and clear. In particular, we now provide detailed explanations of key components of the ship, such as the intercept, mainsail, mast, port and starboard sides, rudder, and engine. These additions will help readers better understand the role and impact of each variable within the model.

Moreover, in the performance analysis section, we have included discussions on how these ship elements influence overall performance and the predictive capabilities of the model.

8. We want to see clear price separation according to region.
**Response 8:** Thank you for your insightful feedback regarding the need for clear price separation according to region. In this section, we have focused our analysis on regional differences by utilizing panel data from 2005 to 2019, specifically dividing the data by geographical areas. Our primary emphasis is on three core sailing markets: the Caribbean, Europe, and the USA.

The specific regional classification and the corresponding data can be found in the accompanying Excel file for reference. To illustrate the price differences more clearly, we have included Table 9, which provides a comparative analysis of prices across the different regions. This table highlights the distinct pricing trends and factors influencing these variations, offering readers a comprehensive view of the market dynamics.

9. We want to see clear price predicted according to year make at each region
**Response 9:** Thank you for your valuable feedback regarding the need for clear predicted prices according to each region and year. In response, we have added a new section, 5.3.3, dedicated to the analysis of regional price predictions.

In this section, we employed the previously discussed price prediction model to test the three major geographical areas: the Caribbean, Europe, and the USA. Our aim was to clearly delineate price differences across these regions over time. The results are presented in Table 9, which details the predicted average prices for each region from 2020 to 2025.

From the predicted prices, it is evident that the USA consistently exhibits the highest average prices, followed by Europe, while the Caribbean reflects the lowest prices. Additionally, the analysis reveals notable trends: the Caribbean region shows significant price fluctuations, Europe displays a downward trend, and the USA experiences relatively stable prices with minor fluctuations.

These findings underscore the regional effects in the second-hand sailing market, as illustrated by the data. By providing clear, year-by-year price predictions for each region, we hope to offer a comprehensive understanding of how pricing dynamics vary geographically over time.

10. Figure 18 does not present a convincing result. The predicted value reflects the overall average price level. Nevertheless, the predicted price here is rendered useless.

Response 10: Thank you for your constructive feedback regarding Figure 18. We appreciate your insights on its effectiveness.

After careful consideration of your comments, we have decided to remove Figure 18 from the manuscript. The figure, which compares the overall average price levels of the two types of sailboats, does not provide substantial value to our research findings and may lead to confusion regarding the model's predictive capabilities.
